# Design of a Vaccine Passport Validation System Using Blockchain-based Architecture: Development Study

**DOI:** 10.2196/32411

**Published:** 2022-04-26

**Authors:** Hsiu An Lee, Wei-Chen Wu, Hsin-Hua Kung, Jai Ganesh Udayasankaran, Yu-Chih Wei, Boonchai Kijsanayotin, Alvin B Marcelo, Chien-Yeh Hsu

**Affiliations:** 1 National Institute of Cancer Research National Health Research Institutes Tainan Taiwan; 2 Standard and Interoperability Lab Smart Healthcare Center of Excellent Taiwan Taipei Taiwan; 3 Department and Graduate Institute of Finance National Taipei University of Business Taipei Taiwan; 4 Sri Sathya Sai Central Trust Prasanthi Nilayam Puttaparthi India; 5 Department of Information and Finance Management National Taipei University of Technology Taipei Taiwan; 6 Department of Clinical Epidemiology and Biostatistics Faculty of Medicine Ramathibodi Hospital Mahidol University Bangkok Thailand; 7 Thai Health Information Standards Development Center Health System Research Institute Ministry of Public Health Bangkok Thailand; 8 University of the Philippines Manila Philippines; 9 Department of Information Management National Taipei University of Nursing and Health Sciences Taipei Taiwan; 10 Master Program in Global Health and Development Taipei Medical University Taipei Taiwan

**Keywords:** COVID-19, vaccine passport, global border control, health policy, international infectious disease strategy, vaccine, policy, strategy, blockchain, privacy, security, testing, verification, certification, Fast Healthcare Interoperability Resource

## Abstract

**Background:**

COVID-19 is an ongoing global pandemic caused by SARS-CoV-2. As of June 2021, 5 emergency vaccines were available for COVID-19 prevention, and with the improvement of vaccination rates and the resumption of activities in each country, verification of vaccination has become an important issue. Currently, in most areas, vaccination and reverse transcription polymerase chain reaction (RT-PCR) test results are certified and validated on paper. This leads to the problem of counterfeit documents. Therefore, a global vaccination record is needed.

**Objective:**

The main objective of this study is to design a vaccine passport (VP) validation system based on a general blockchain architecture for international use in a simulated environment. With decentralized characteristics, the system is expected to have the advantages of low cost, high interoperability, effectiveness, security, and verifiability through blockchain architecture.

**Methods:**

The blockchain decentralized mechanism was used to build an open and anticounterfeiting information platform for VPs. The contents of a vaccination card are recorded according to international Fast Healthcare Interoperability Resource (FHIR) standards, and blockchain smart contracts (SCs) are used for authorization and authentication to achieve hierarchical management of various international hospitals and people receiving injections. The blockchain stores an encrypted vaccination path managed by the user who manages the private key. The blockchain uses the proof-of-authority (PoA) public chain and can access all information through the specified chain. This will achieve the goal of keeping development costs low and streamlining vaccine transit management so that countries in different economies can use them.

**Results:**

The openness of the blockchain helps to create transparency and data accuracy. This blockchain architecture contains a total of 3 entities. All approvals are published on Open Ledger. Smart certificates enable authorization and authentication, and encryption and decryption mechanisms guarantee data protection. This proof of concept demonstrates the design of blockchain architecture, which can achieve accurate global VP verification at an affordable price. In this study, an actual VP case was established and demonstrated. An open blockchain, an individually approved certification mechanism, and an international standard vaccination record were introduced.

**Conclusions:**

Blockchain architecture can be used to build a viable international VP authentication process with the advantages of low cost, high interoperability, effectiveness, security, and verifiability.

## Introduction

### Background

COVID-19 is an ongoing pandemic caused by SARS-CoV-2. The disease was discovered at the end of 2019 [1] in Wuhan City, Hubei Province, People's Republic of China, which rapidly spread to many countries worldwide in early 2020 and eventually became a global pandemic. More than 170 million confirmed cases had been reported in countries and regions worldwide as of June 2021, with over 3.7 million deaths [2-4], making COVID-19 1 of the largest epidemics in human history. There are at least 287 COVID-19-preventive vaccines available in the world [5], with 5 vaccines (Pfizer/BioNTech, Astrazeneca-SK Bio, Serum Institute of India, Janssen, and Moderna) available for emergency use in June 2021, but none have completed clinical trials. Nonetheless, as vaccines are developed, disease conditions in various countries are gradually being controlled.

Currently, all vaccination or reverse transcription polymerase chain reaction (RT-PCR) test results are certified and verified on paper by hospitals or testing institutions. Meanwhile, falsified documents pose a significant risk in confirming vaccines or conducting testing. At the end of 2020, the European Union (EU) began discussions on the design of vaccine passports (VPs). The official policies and measures were announced in June 2021 and went into effect in July 2021. The EU Digital COVID Certificate is available in both digital and paper formats, and it includes an official quick response (QR) code to ensure its authenticity. As a result, Japan began to design VPs as well. Although, based on the current strategy, the VP may become an important key to future global activities, the problems of document verification and data use among countries have yet to be resolved.

The Vaccination Certificate Program (VCI) is a consortium of 12 information technology and health care organizations, including Microsoft, Oracle, and the Mayo Clinic [6-8]. The project's goal is to provide a secure and decentralized solution for open licenses that is interoperable and widely adopted across multiple platforms. The software they proposed enables the exchange of verifiable clinical data via smart health cards or digital health wallets. As a result, it will require an ecosystem of interoperable applications, data, and processes. The VCI provides digital access to vaccination records by utilizing smart health cards open specifications based on W3C Verifiable Credential and Health Level Seven International (HL7) Fast Healthcare Interoperability Resource (FHIR) standards. The VCI solution will necessitate the use of a token (smart card) and membership in an alliance, both of which are not required in our blockchain architecture.

Our team proposed “a blockchain-based global infectious disease surveillance and case-tracking model for COVID-19” [9] by the end of 2020. Although the primary goal of the COVID-19 defense was to prevent the virus from spreading across borders, we demonstrated how the blockchain can help with defense and ensure that information is correct. Another study conducted by our team demonstrated the viability of blockchain-based global information exchange [10,11]. Gstrein [12] proposed using blockchain to establish immunity certificates. The authors also suggested using immutable blockchain technology to prevent the spread of fraudulent information and reports. Furthermore, this research tries to address the issues of candidate privacy and anonymity.

### Objectives

The worldwide VP verification mechanism is currently incomplete. There is no universal standard for digital VPs, and paper VPs are suspect of fabrication. Many developing nations cannot afford the worldwide data integration structure, since it requires a large number of resources and money. To address these issues, this study provides an innovative study of a global architecture that uses blockchain to complete the verification and distribution of VPs, such as RT-PCR testing results or vaccination records. The blockchain's public verification is utilized for passport verification, while smart contracts (SCs) with various purposes verify that a user's VP is valid. Finally, the public chain technique can be used to save building costs and create a worldwide VP chain.

## Methods

### Study Design

**Figure 1:**
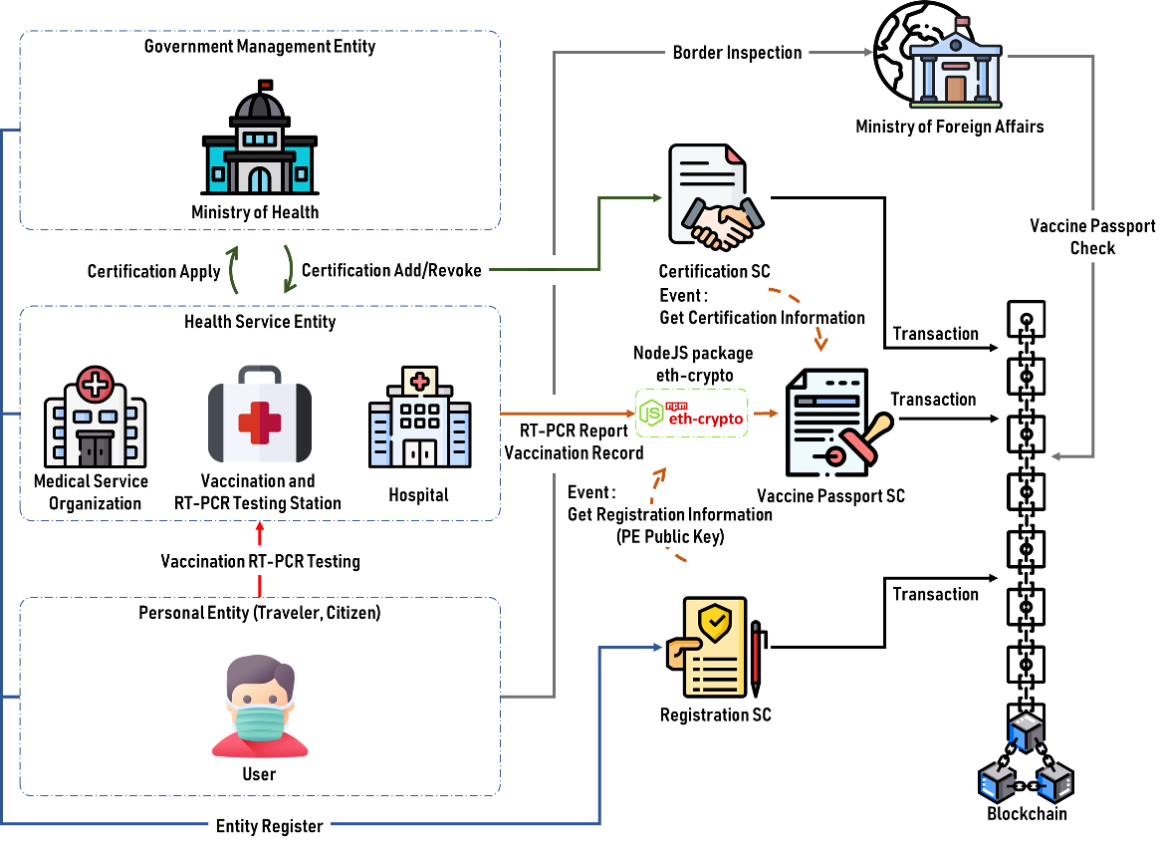
Overview of the blockchain architecture for VPs. PE: personal entity; RT-PCR: reverse transcription polymerase chain reaction; SC: smart contract; VP: vaccine passport.

The architectural design application is divided into 2 parts: entity architecture and blockchain architecture. Basically, the system is an architecture of multientities interacting with each other using SCs built on the blockchain. Government-managed entities (Ministry of Health [MoH]), health service entities (eg, medical service organizations, medical stations, and hospitals), personal entities (eg, passengers, citizens, and patients), and border control entities (BCEs) are all classed according to their duties (Ministry of Foreign Affairs). The blockchain includes SCs and the public chain. Individual functions are discussed later. An overview of the blockchain architecture for VPs is shown in Figure 1.

### Entities’ Identification and Application Process

#### Government-Managed Entity

In this study, a government-managed entity (GME) is a government agent that issues certificates to health service organizations to identify that the vaccination certificates or testing results submitted by the organizations can be trusted at border inspections. In addition, the identification of a GME, including the MoH, the Centers for Disease Control and Prevention (CDC), and the COVID-19 Central Command Center, is recognized by every country's government.

#### Health Service Entity

A health service entity (HSE) is described as a facility that can provide a VP. Medical service organizations, medical stations, and hospitals are all included. Every HSE should request a certificate from the local GME to demonstrate that the former's reports can be trusted. The certificate will then be recorded in the blockchain through the certificate SC. When a personal entity (PE) accepts the HSE vaccine or RT-PCR testing services, the latter should get the PE's blockchain address. After the VP is completed, it is encrypted using *eth-crypto*, a Node.js-supported encryption software program. The encrypted VP is then recorded in the blockchain via the VP SC. The complete procedure is outlined later.

#### Personal Entity

A PE is described as a normal person seeking a VP via vaccination or RT-PCR testing in an HSE. When a PE registers on the blockchain via the SC, their blockchain address is generated. The identity of a PE includes travelers, citizens, patients, and others who want to get a VP. When a PE obtains a vaccine or RT-PCR testing from an HSE, the PE must supply the HSE with a blockchain address. Upon completion, the VP is transferred into the blockchain through the VP SC. The private key of the PE can then be used to decode the VP contained in the chain.

#### Border Control Entity

A BCE is defined as the unit in each country responsible for border control. The Ministry of Foreign Affairs is part of a BCE's identity. During border inspections, a BCE gets encrypted VPs of PEs from the blockchain, where the PEs are asked to provide a private key to decrypt their individual encrypted VPs. The problem of falsified documents is handled because all VPs come from the blockchain and are provided by verified HSEs. Furthermore, countries can reach an agreement to allow BCEs to accept validated VPs from various HSEs as long as the HSHs are certified. The public chain is used by this framework to facilitate interoperability. The BCE does not require blockchain registration, since the BCE only needs to retrieve data. Additionally, in this multientity architecture, any entity can become a BCE in order to authenticate a PE's VP.

### Vaccine Passport Content and Format

In this study, the World Health Organization’s (WHO) interim guidelines for establishing a Smart Vaccination Certificate release was followed [13-16]. Vaccination information, the vaccination hospital, date, time, and the RT-PCR testing report should all be included in the VP. Only a certified or recognized HSE can complete a VP. In addition, the FHIR standard format was used in this research. In recent years, the FHIR has become the most widely used medical data standard and framework. FHIR adoption can increase data interoperability and usability, allowing users to quickly integrate the VP blockchain into their systems.

In the FHIR architecture, various resources are used to integrate the entire VP, which include patient (PE), organization (HSE), practitioner (administrative or medical staff), immunization (first or second dose injection records), immunization recommendation (second dose of vaccine reservation information), and observation (RT-PCR/quick screening and testing report). The FHIR resource structure of the VP is provided in Multimedia Appendix 1.

### Blockchain Architecture and the Functions of a Smart Contract

#### Blockchain Architecture

To reduce resource consumption and VP usage restrictions, this study used the proof-of-authority (PoA) consensus technique, which allows authorized nodes to join as block-establishing nodes. The number of nodes in the PoA consensus process is unlimited, but the number of validators is limited. The nodes are in charge of synchronizing the blockchain ledger, while the verifier is in charge of confirming transactions and packaging blocks. The blockchain of the PoA consensus method has the potential to outperform conventional and decentralized public chains, such as Bitcoin and Ethereum, in terms of efficiency and scalability, due to the limited number of validators.

Authorized nodes are set as government agencies of various countries, while countries worldwide jointly maintain the verification of blocks. The blockchain architecture was Ethereum's public chain, and the Ethereum protocol was used to move VPs to the blockchain architecture, create a new block, and connect it to the blockchain using Geth (Go Ethereum). Due to the regulations of different countries or organizations, it is acceptable if certain BCE-permitted VPs for PEs are created by an HSE that has not obtained a certificate from a GME.

#### SCs of Different Functions

A total of 3 SCs are used in the research architecture: registration, VP, and certification SCs.

The registration SC is used to store the public keys of all users, which are used to encrypt VPs.

The VP SC is used by HSE to generate VPs, including VP encryption and storage of HSE certification information.

The certification SC is used to issue the certification authority of the organization, and the main purpose is that each country can develop and certify its own recognized HSE. When the BCE checks the blockchain VP information, it can quickly confirm the HSE certification.

## Results

In the scenario, there are 3 entities: a PE seeking a VP, an HSE who can administer vaccinations, and a GME representing the MoH.

### VP Verification

**Figure 2:**
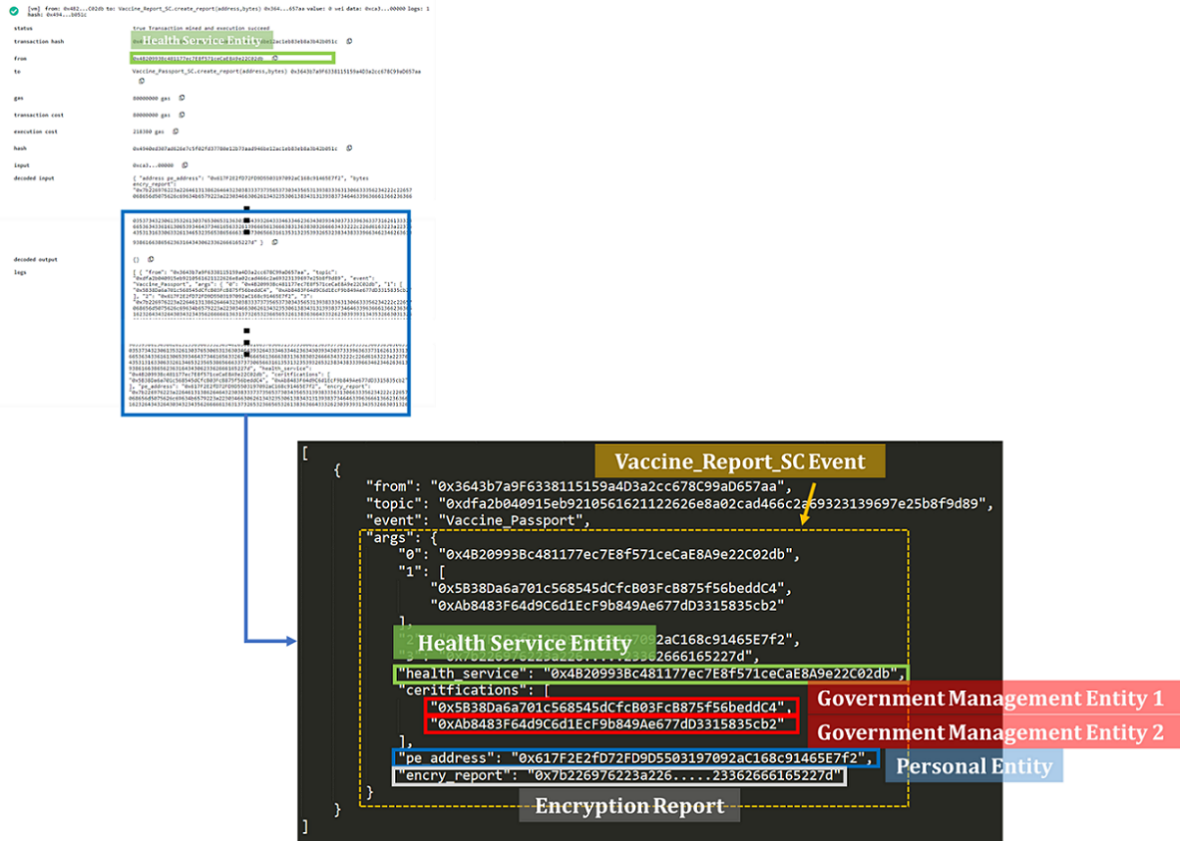
The blockchain content of a VP. VP: vaccine passport.

A PE wants to travel to other countries. The BCE system uses the blockchain to retrieve the encrypted VP. The PE's private key is required to decrypt the VP after passing HSE authentication. After that, the PE goes through VP verification to pass the border check. Figure 2 shows the PE's VP data recorded in the blockchain, which includes the HSE, HSE certificate, PE, and encrypted VP.

After obtaining the block data, the PE should provide a private key to unlock the encrypted VP. Then, the decrypted VP is presented in FHIR format, which can be read through the front-end graphical user interface (GUI).

#### VP Content and International Standard

**Figure 3:**
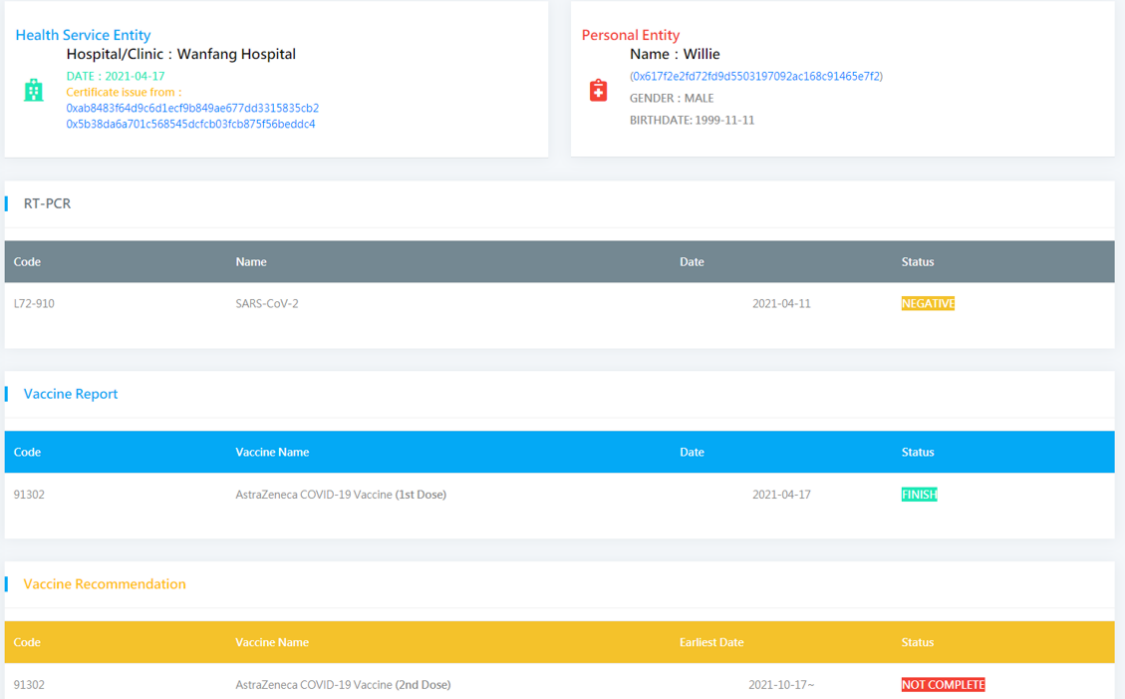
The GUI of an electronic VP for border inspection. GUI: graphical user interface; RT-PCR: reverse transcription polymerase chain reaction; VP: vaccine passport.

In this study, the VP contained (1) patient information (eg, name, national ID number/residence permit number/passport number, biological sex, nationality, birthday), (2) vaccine information (eg, vaccine type, vaccine brand, manufacturer, number of doses, vaccination date, name/code of the medical institution responsible for vaccination, name/code of the medical staff who administered the vaccine, country where the vaccination was given, date of next vaccination record), and (3) RT-PCR test reports (eg, disease name [COVID-19 or SARS-CoV-2], inspection date, report output date, testing method [needs to be tested by molecular biology nucleic acid], and check report results, including negative, positive, and undetectable). To verify the VP, the inspection unit should double-check its content and the information obtained in the block. The VP presented is shown in Figure 3.

#### Testing Feedback From a Different Country’s User

This study proposed a blockchain architecture for the authentication process of an executable international VP, with the advantages of low cost, high interoperability, effectiveness, security, and verifiability. In Southeast Asian countries, a regional travel protocol has been designed and cross-nationally tested. The findings reveal that the proposed architecture is capable of quickly verifying the VP for international travel.

The international standard format of the HL7 FHIR was designed in this architecture to ensure interoperability. The BCE can easily obtain a traveler's validated VP, which has been verified by the source country's supervisory unit.

Testing was performed on a simulated public chain environment. A total of 3 testing scenarios were tested in this study. In the testing process, 3 identities were registered. Both the certification SC and the VP SC required the signature of the private key. The private key was also used to decrypt the VP stored on the chain. All the users can use the web-based GUI shown in Figure 3.

The testing process was designed to simulate the scenario of a traveler using a VP. The simulation process was as follows:

1. The GME (country X MoH), the HSE (hospital A), and the PE (traveler) register a VP blockchain account.
2. Hospital A applies for authorization from the country X MoH through an SC.
3. The country X MoH announces the blockchain account to foreign BCEs for foreign verification of the permission (from the country X MoH) of hospital A.
4. The traveler goes to hospital A receiving vaccination and provides the blockchain account to hospital A.
5. Hospital A produces a VP for the traveler after vaccination and uploads the traveler's VP to the VP blockchain via the SC. The traveler receives an index of the VP on the blockchain.
6. The traveler travels abroad and provides the index of the VP to the foreign BCE.
7. The BCE obtains encrypted VP data.
8. The traveler provides a private key (obtained at step 1) for VP decryption.
9. The BCE confirms the VP content and the authorization of hospital A.
10. The verification is completed, allowing the traveler to enter the country.

**Figure 4:**
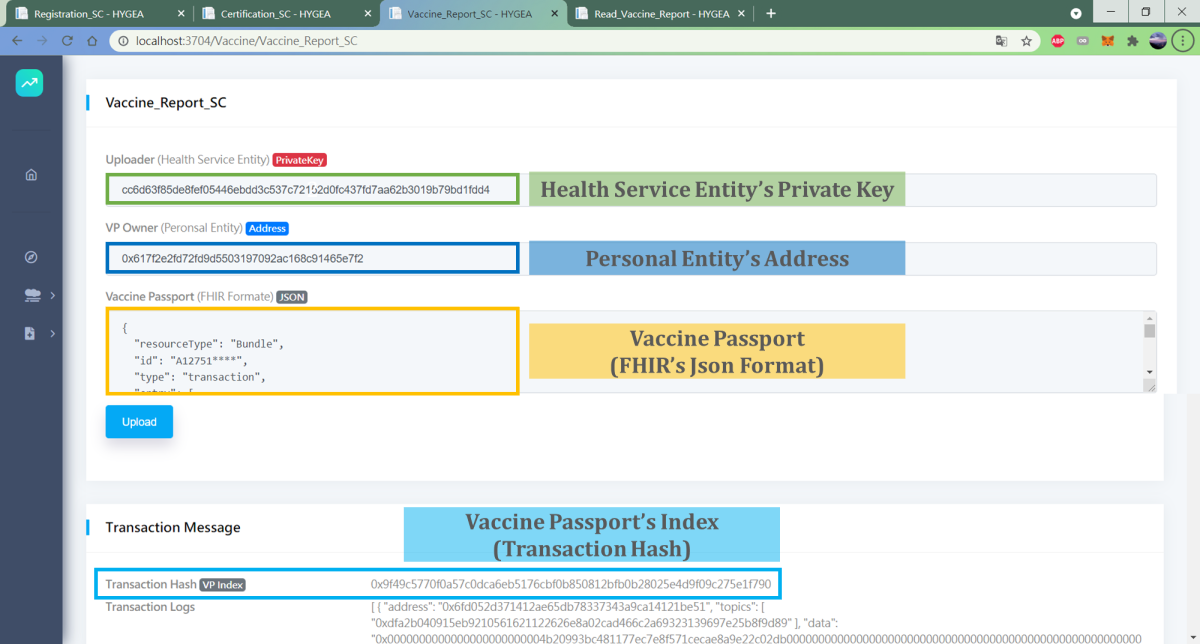
The GUI of VP production. FHIR: Fast Healthcare Interoperability Resource; GUI: graphical user interface; HSE: health service entity; VP: vaccine passport.

**Figure 5:**
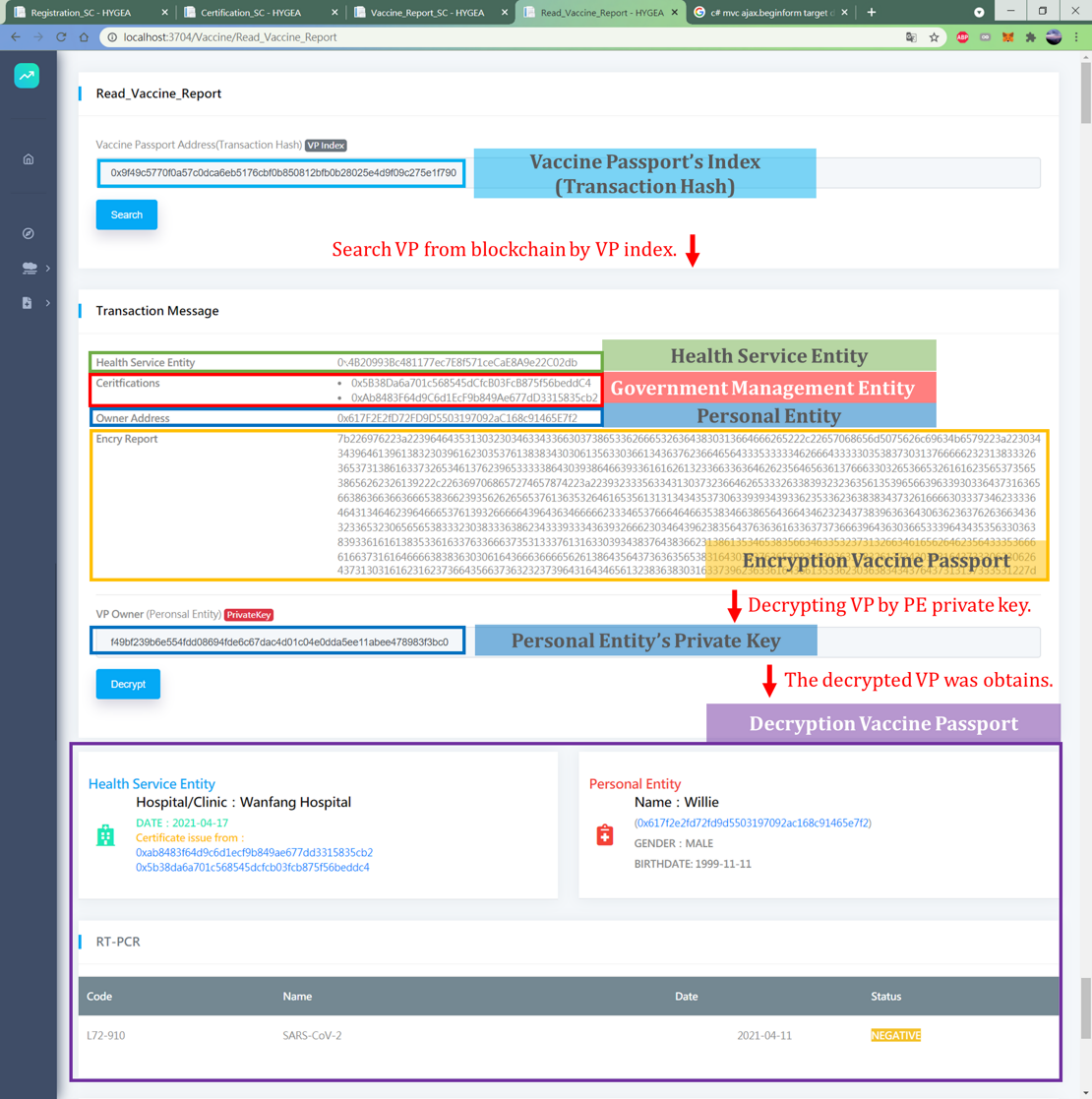
The GUI of VP decryption and VP content. GUI: graphical user interface; PE: personal entity; RT-PCR: reverse transcription polymerase chain reaction; VP: vaccine passport.

The GUI of step 5 (VP production) is shown in Figure 4. The GUI of steps 7-9 is shown in Figure 5.

In the first scenario, this was performed according to our test process. A web-based user portal was used to present the contents of the VP, as shown in Figure 3. The data of the VP were encrypted and stored in a block, which needed to be decrypted using the private key of the PE.

In the second scenario, we attempted to create a fake VP. With the design of the SC and decryption mechanism, if the PE tries to authenticate with another person's VP, the encrypted VP will not be decrypted due to a private key error.

In the third scenario, we tested VPs generated by the HSE that have not obtained any GME certificates. When decrypting such VPs, the hospital certificate field is emptied. Therefore, the VP is considered an invalid VP, when checked.

The platform is currently in its testing stage, and there is a small number of users on the architecture chain. The users’ comments are summarized as follows:

- Blockchain architecture helps to verify the correctness of data.
- The construction cost of the blockchain architecture is low, and it can be quickly deployed and expanded to local areas.
- The protection provided by blockchain technology can make users believe that the system is secure.
- VP-issuing units and verification units can be clearly displayed in the VP to improve the safety of border control.

#### Efficiency and Cost of Public Blockchain Architecture

In the traditional authentication mechanism, facilities such as servers, users, and encrypted secure networks are required. The cost includes a hardware environment, software platform development, and personnel maintenance costs. However, the blockchain architecture under the PoA architecture can save these costs (Table 1).

In this study, a set of blockchain test environments was constructed for comparison.

The PoA blockchain is built on 3 basic computers (because it is PoA, it does not require supercomputing power). The BCE needs a general internet connection to obtain the encrypted VP on the blockchain. The user only needs to provide the private key to decrypt the personal VP.

**Table 1:** The cost comparison table between blockchain and traditional architectures.

Facility	Blockchain-based architecture	Traditional architecture
Server	3 general servers	All VP^a^ providers must have advanced servers (each country at least 1 server).
Internet	General internet	High-security protection.
System maintainer	At least 1 for each service server	At least 1 for each service server.

^a^VP: vaccine passport.

## Discussion

### Principal Findings

The major goal of this study was to design a general VP verification architecture for international use. The decentralized mechanism of the blockchain was used to build an open and unforgeable VP information platform. The blockchain architecture proposed in this research is a single standardized vaccine verification system framework that can be jointly created by the public and private sectors. The content of the VP is recorded using the FHIR international standard, and the SC of the blockchain is used for authorization and authentication to achieve hierarchical management of different international hospitals and individuals who receive the injection. The blockchain stores the encrypted VP, which is managed by the user who manages private keys. The blockchain uses the public chain of the PoA, and all information can be accessed on the designated chain, achieving the goal of low development cost and high efficiency in VP management, so countries in different economic states can use it. Additionally, the vaccine holder only needs to bring the private key—text, barcode, or cold wallet document—to complete the VP verification. This free authentication mechanism convinces government agencies, border control, airlines, hotels, department stores, restaurants, educators, and others to believe that these data are authentic and reliable and can design different authentication procedures to issue authorization verification certificates freely.

The FHIR is a standard issued by HL7. It evolved from the HL7 v2, HL7 v3, and HL7 Clinical Document Architecture (CDA) standards and aims to be easier to implement [1]. However, the interoperability between different systems in the medical field is poor. There are many problems that need to be solved for system interoperability. HL7 developed the FHIR as the basis for achieving interoperability. The FHIR is a widely used health information standard that describes the data format and data elements of electronic health records (EHRs). Schleyer et al [2] developed an FHIR-based medical dashboard that integrates clinical data and EHRs from the hospital information system (HIS). The test results after use by medical institutions show that the integrated dashboard is useful. Based on the FHIR, clinical information can be effectively integrated [2]. The FHIR uses a widely adopted network technology, is friendly to the same standard system and independent of users, and provides useful applications and format frameworks for EHR providers, health care providers, and public health [3]. Recently, many studies and industry products have demonstrated how the FHIR can be used for health care data integration [4-11] and have discussed how the FHIR can achieve interoperability between different health care systems.

Public and private organizations worldwide are looking for ways to fight COVID-19 in the hopes of finding a strategy and solution that can help society return to “normal.” Vaccination is the most critical issue at present. When herd immunity is achieved, what follows is how to restore “normal” activities worldwide.

The political issues and policies of different countries create problems when exchanging VPs between them. For example, the EU Digital COVID Certificate Regulation [12] was available since July 1, 2021. EU residents and citizens can have their EU Digital COVID Certificates in QR code or paper format issued and verified across the EU countries. That is the first solution proposed to prove an individual’s vaccination status. Presently, the United States uses handwritten paper certificates as proof of vaccination. Some states also have digital passes, but they have not constructed a complete verification and international use strategy. However, although paper documents are usually illegible or incorrect, they are also easy to lose and forge. Moreover, the mobile app–based authentication system itself is not complete, proving ineffective on a global scale amidst the majority’s lack of access to smartphones.

These verification certificates can allow organizations to plan on their own, such as identifying which organization accepts the VP, who has been certified by the organization, and whether the VP is authentic and has not been revoked. Therefore, the blockchain and public ledgers can be used as the frontline application of the VP verification system. If properly operated, the public ledgers cannot be forged and can reliably [13] prove an individual’s vaccination status.

The blockchain was originally used in the medical field and faced challenges related to transparency and confidentiality because “everyone can see everything” on the blockchain network [14-16]. The increasing transparency and decline of confidentiality, such as information on transmissions, are generally considered limitations of the blockchain. However, this limitation is an advantage in the process of using VPs. As VP holders need to prove to everyone that they have been vaccinated, such change gives the application of the blockchain a good advantage and the blockchain can be quickly deployed at low cost. Furthermore, different application SCs are developed in this research framework. Through SCs, entities of different levels can make individual authentication policies, which greatly improves usability and interoperability. Furthermore, cryptocurrency is inherently contained in the blockchain VP system of our design. If there is a need for payment for VP exchange and verification, the function of payment is available in our system.

### Future Directions

By comparison with prior work, there are several important points that need to be studies in the future. First, blockchain technology ensures data security and privacy and has been successfully used in different domains. However, more study is needed to demonstrate the benefits of using the blockchain for VPs for international users. Second, for the blockchain architecture of VPs to work smoothly, it is preferred that a cross-country VP alliance service system be developed for international C0VID-19 defense.

### Conclusions

Although many countries endeavor to reopen their economies and allow inward and outward tourism, there is still uncertainty on the state of health of citizens from different countries with varying degrees of policies and types of vaccination. The use of VPs is 1 of the ways to promote the recovery of global activities. Unfortunately, at present, VPs are still difficult for many countries to implement. The verification, trust, and effectiveness of paper documents must be considered. The simple blockchain architecture proposed in this research can be implemented collaboratively by public and private entities and be rapidly expanded. The open nature of the blockchain contributes to establishing transparency and data accuracy. The smart certificate enables authorization and authentication, while the encryption and decryption mechanism ensures data protection. To make it globally available and accessible, the FHIR international data standard was adopted in this research.

The principal findings in this study are as follows: First, blockchain architecture was used to build the authentication process of an executable international VP, with advantages of low cost, high interoperability, effectiveness, security, and verifiability. Second, the international data standard FHIR was adopted in this research. Third, this PoA demonstrated the design of blockchain architecture that, when adopted, can accurately achieve global VP verification and at a cost any country can afford. Fourth, the platform has been tested by several users in different countries in the Asia eHealth Information Network (AeHIN) and has shown that it is a suitable platform for VP verification.

## Appendix

Multimedia Appendix 1 The FHIR resource structure of a VP, encrypted VP contents, and functions of an SC in a blockchain. FHIR: Fast Healthcare Interoperability Resource; SC: smart contract; VP: vaccine passport.

## Abbreviations

BCE: border control entity
EU: European Union
FHIR: Fast Healthcare Interoperability Resource
GME: government-managed entity
GUI: graphical user interface
HL7: Health Level Seven International
HSE: health service entity
MoH: Ministry of Health
PE: personal entity
PoA: proof of authority
QR: quick response
RT-PCR: reverse transcription polymerase chain reaction
SC: smart contract
VCI: Vaccination Certificate Program
VP: vaccine passport
