# Supplementary material for: Design of a Vaccine Passport Validation System Using Blockchain-based Architecture: Development Study
Source: JMIR Public Health Surveill. 2022 Apr 26;8(4):e32411. doi: 10.2196/32411 (PMC9045485; doi:10.2196/32411)
Supplement: Multimedia Appendix 1 [file publichealth_v8i4e32411_app1.docx]

**Multimedia Appendix 1.** The FHIR resource structure of a VP, encrypted VP contents, and functions of an SC in a blockchain. FHIR: Fast Healthcare Interoperability Resource; SC: smart contract; VP: vaccine passport.

1. **The FHIR resources structure of VP**


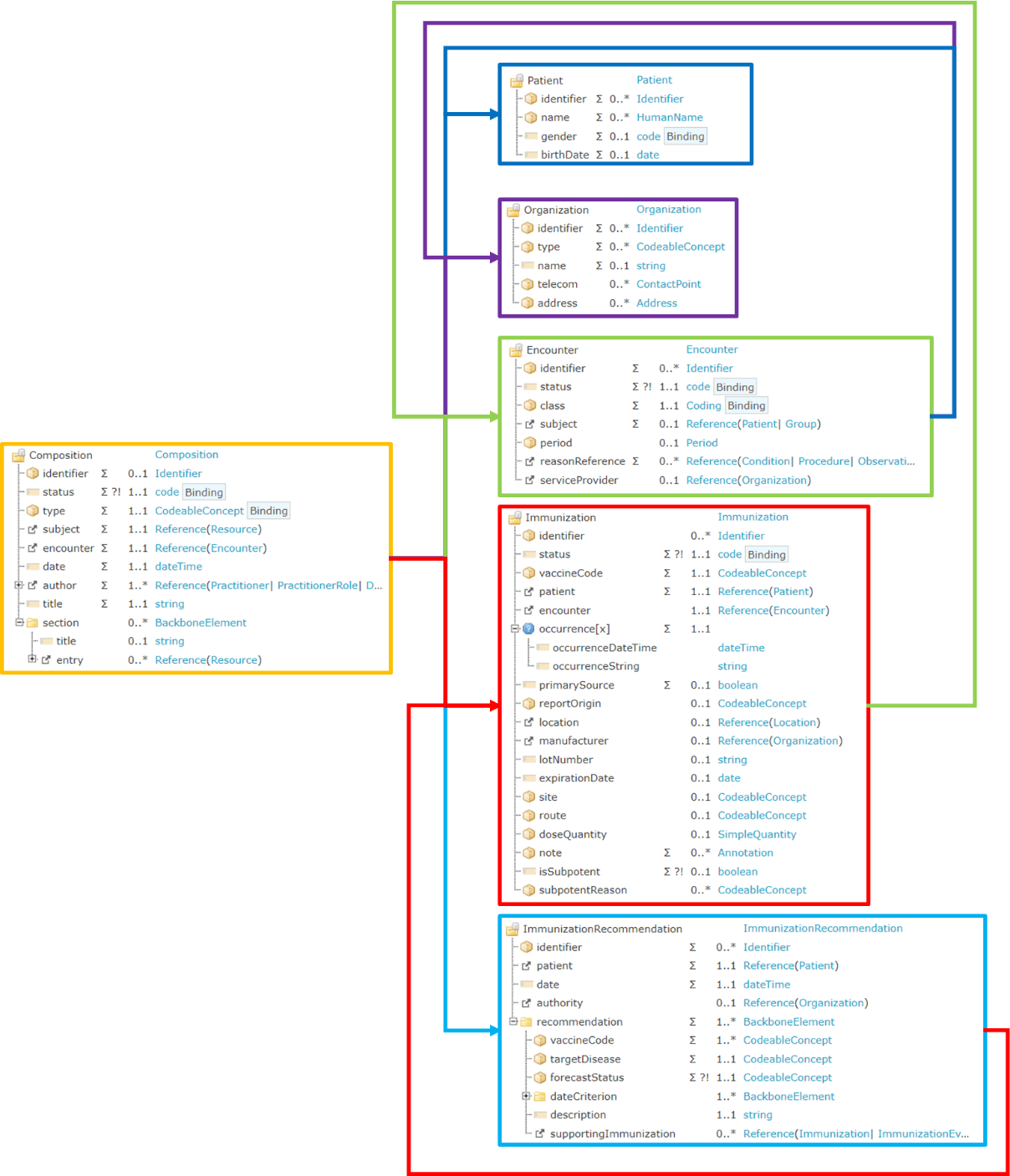


1. **The encrypted VP contents**


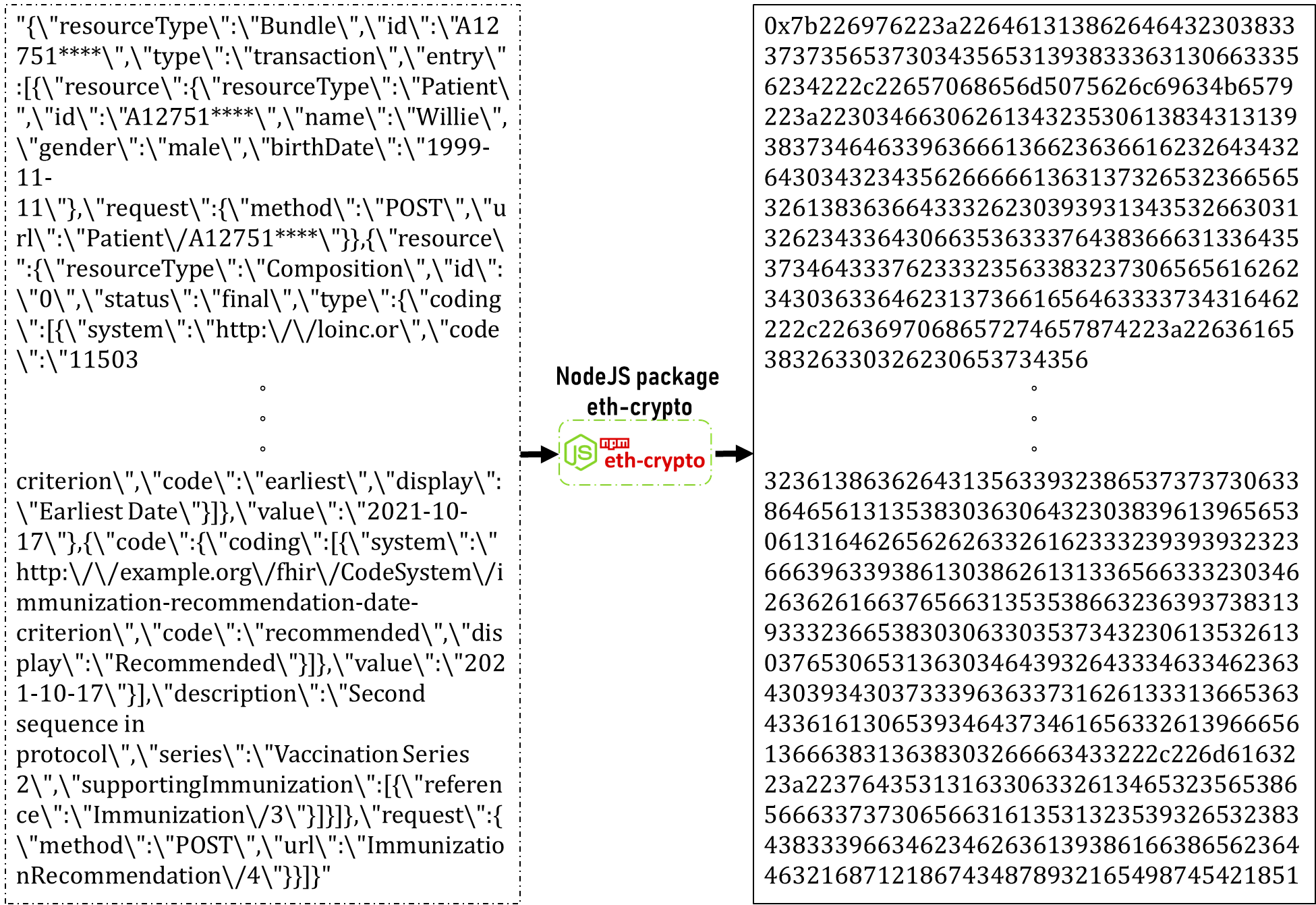


1. **Functions of Smart Contract in Blockchain**
2. Registration Smart Contract

When a PE, HSE, or GME submits a registration request, the registration SC generates an account address and a set of asymmetric public and private keys using Algorithm 1. The address is used to link to the PE's blockchain account. On the other hand, the public key is used to encrypt a PE’s VP data. Both the address and the public key are stored in a smart contract event. The private key used to decrypt the encrypted VP stored in the blockchain will be provided to the PE for safekeeping.


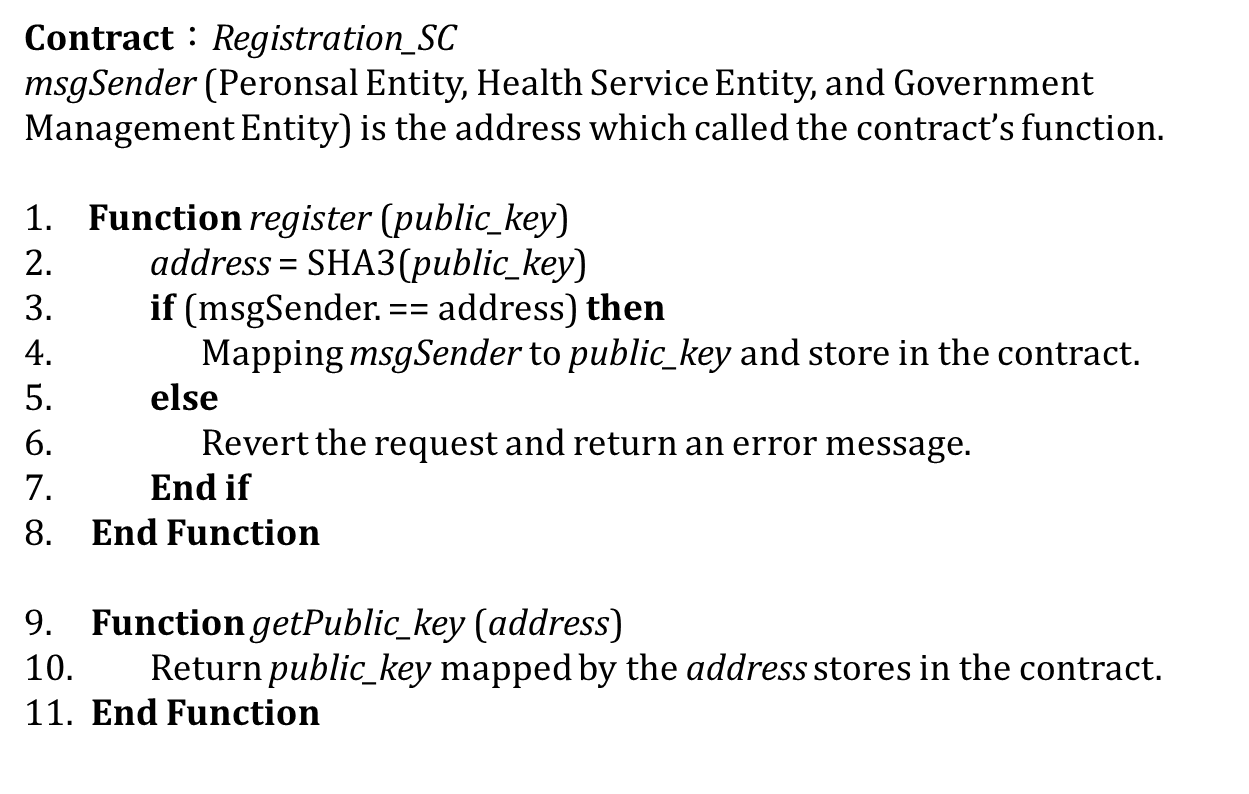


Algorithm 1 Blockchain Smart Contract – Registration SC

All three entities must create an account through the registration SC. Figure 1 shows the registration SC information. A PE, an HSE, and two GMEs were among the four entities registered in our demo.


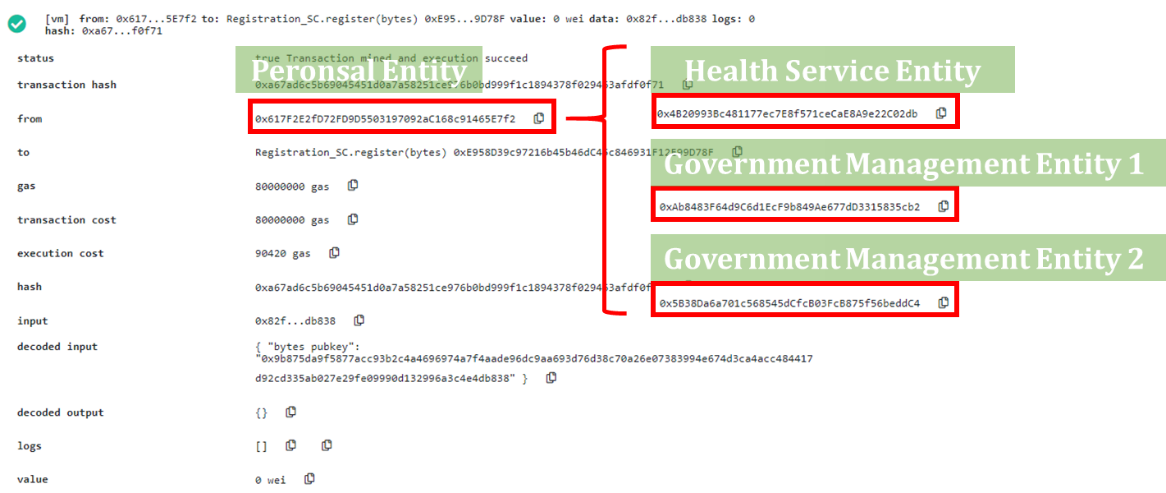


Figure 1 Registration Smart Contract transaction information

1. Vaccine Passport Smart Contract

The smart contract is used to transfer the VP to the blockchain using Algorithm 2. The HSE should connect to the encryption API "eth-crypto" to perform the VP encryption process once the VP has been produced. The encryption API receives the PE's public key from the registration SC. In the encryption API, the Rivest–Shamir–Adleman (RSA) public-key cryptosystem is used for PE's VP encryption. The VP SC receives the encrypted VP generated following the encryption procedure, as well as the HSE certificate from the certification SC. The encrypted VP, HSE address on the blockchain, the unit that issued the certificate to the HSE, and the time the VP was generated will all be included in the transaction.


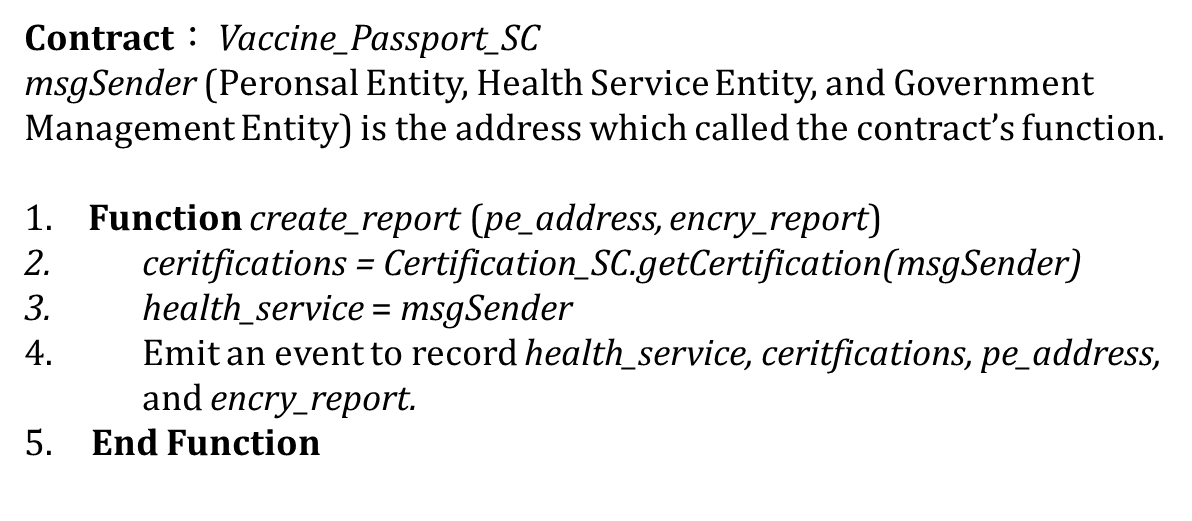


Algorithm 2 Blockchain Smart Contract – Vaccine Passport SC

The workflow of VP SC as following:

Step 1: PE's vaccine passport is created by HSE.

Step 2: HSE initiates transaction with PE through vaccine passport SC.

Step 3: Encrypted PE's vaccine passport by PE's public key through RSA.

Step 4: SC connects to registration SC and certificate SC gets the information.

Step 5: Integrates data, including Encrypted PE's vaccine passport, HSE address, certificate information, and vaccine passport generated time.

Step 6: A block is created.

A PE visits an HSE to get vaccinated or have RT-PCR testing done in order to get a vaccine passport. An HSE initiates the VP transactions to the PE through the blockchain when the VP is completed. The HSE should use the FHIR format to generate the VP, which will be encrypted by the encryption API. After the encryption procedure, the encrypted VP of a PE is placed in the blockchain. Figure 2 shows the VP SC transaction information.


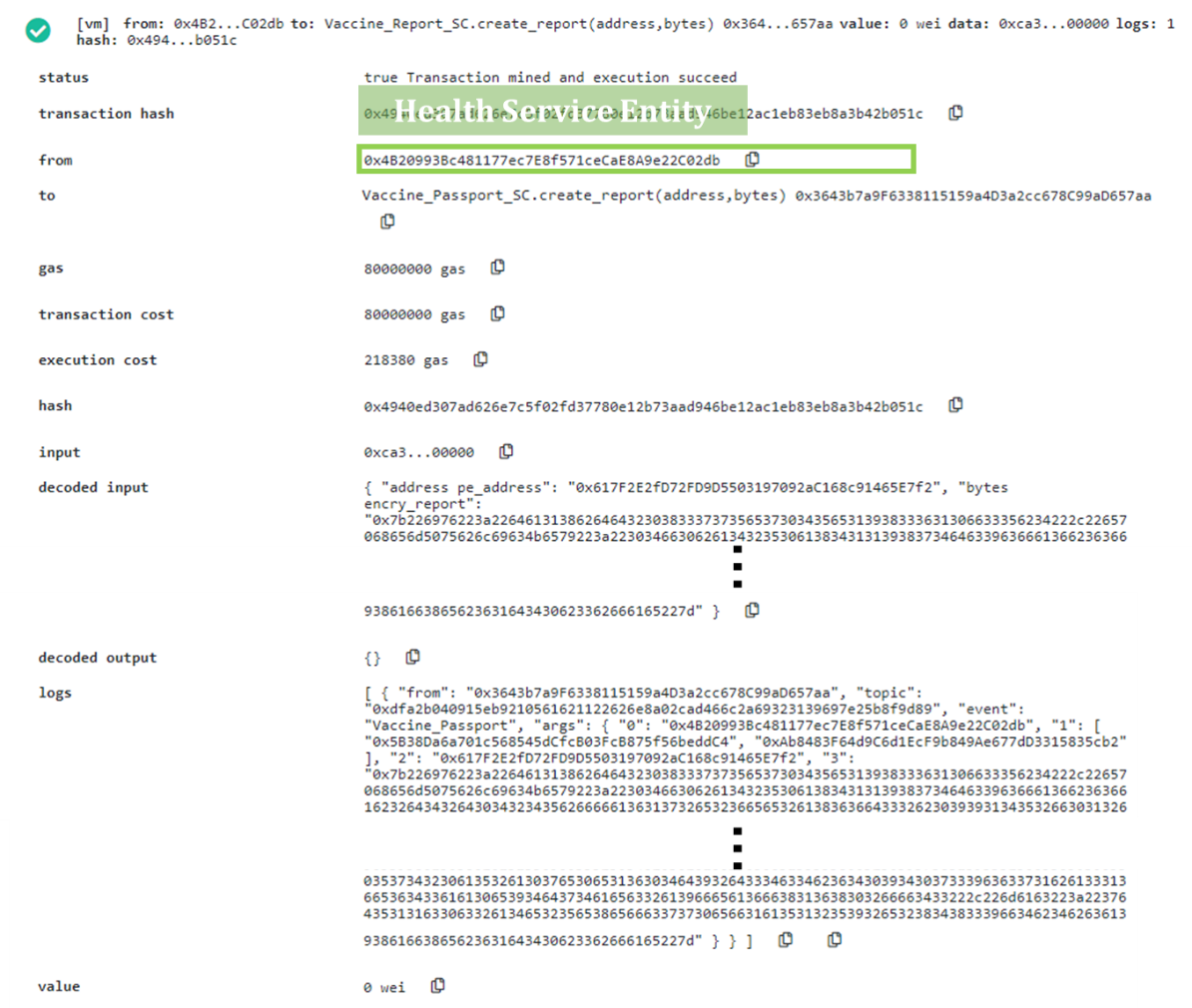


Figure 2 Vaccine Passport Smart Contract transaction information

1. Certification Smart Contract

The certification SC aims to improve interoperability by allowing any entity to issue certificates to other entities. Furthermore, the approval of a certification unit shall be decided by a BCE. For example, the certification accepted by a national-level BCE should be the HSE proposed by the Ministry of Health and Welfare. However, if it is an enterprise-level BCE, it may be possible to accept a VP from other inspection or designated institutions according to their strategies. Such a structure can allow more units to join the application and expand the scope of protection functions of the entire blockchain. Simultaneously, businesses, department stores, restaurants, and other places where people gather can be added to the program, enhancing the efficiency of infectious illness prevention. All entities in the blockchain can provide authentication to other entities using Algorithm 3. Each blockchain entity will have a representative address, which the SC will record in the event list if one entity certifies another. When the VP verification is in progress, the certified address can be used to determine whether the BCE has obtained the certification of the designated unit.


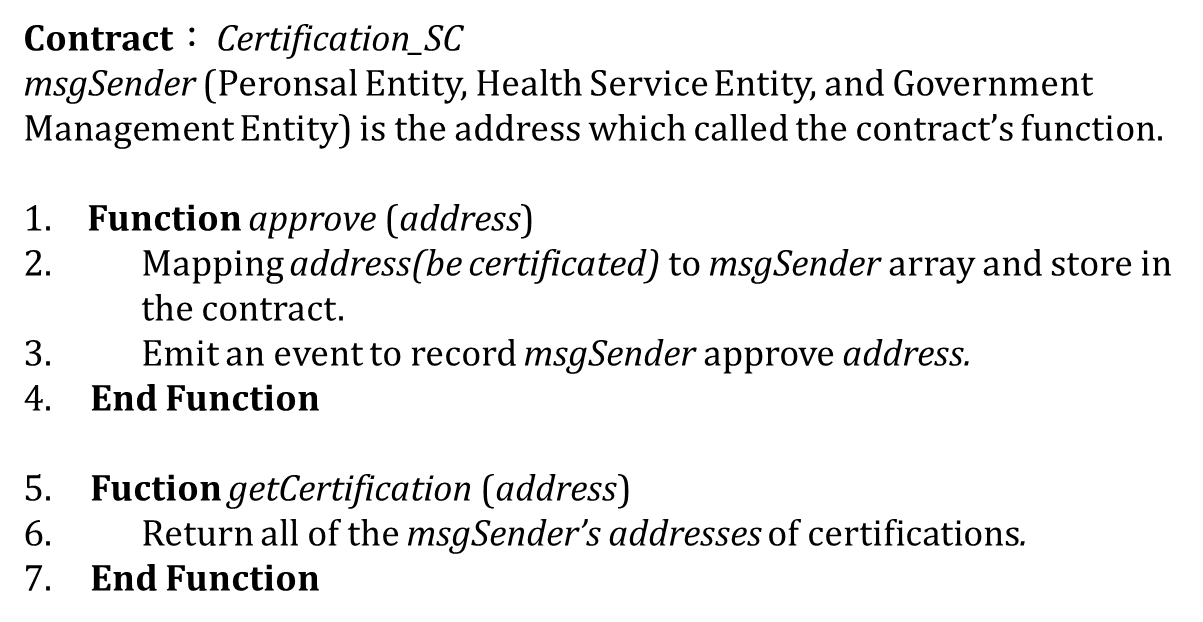


Algorithm 3 Blockchain Smart Contract – Certification SC

An HSE receives certificates from a GME. Every entity has the ability to issue certificates to other entities. Two GMEs are granting certificates to the same HSE in this demonstration. Figure 3 shows the information certification SC.


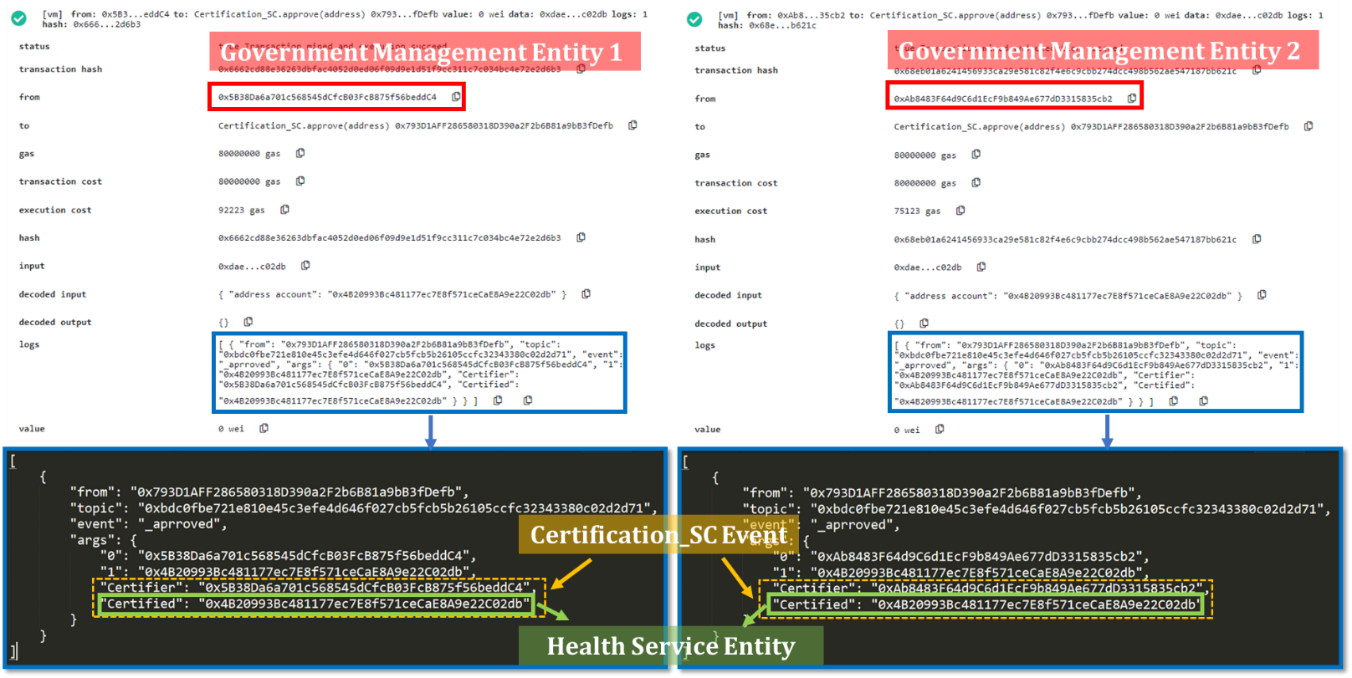


Figure 3 Certification Smart Contract transaction information
